# Geschlechtsspezifische Unterschiede in Ergebnis und Überleben bei der Frühzystektomie: Eine systematische Übersichtsarbeit

**DOI:** 10.1007/s00120-025-02665-8

**Published:** 2025-08-05

**Authors:** Laila Schneidewind, Bernhard Kiss, Nicolas Arnold, Jennifer Kranz, Friedemann Zengerling, Annabel Spek, Thomas Neumann, Annemarie Uhlig

**Affiliations:** 1 Universitätsklinik für Urologie, Inselspital Bern, Wilhelm-Fabry-Haus, Universität Bern, Freiburgstr. 37, 3010 Bern, Schweiz; 2 Klinik für Urologie und Kinderurologie, Uniklinik RWTH Aachen, Aachen, Deutschland; 3 Universitätsklinik und Poliklinik für Urologie, Universitätsklinikum Halle (Saale), Halle (Saale), Deutschland; 4 Zentrum für Integrative Onkologie (CIO), Uniklinik RWTH Aachen, Aachen, Deutschland; 5 Klinik für Urologie und Kinderurologie, Universitätsklinikum Ulm, Ulm, Deutschland; 6 Urologische Klinik und Poliklinik, LMU München, München, Deutschland; 7 Klinik für Hämatologie/Onkologie, Universitätsmedizin Greifswald, Greifswald, Deutschland; 8 Klinik für Urologie, Universitätsmedizin Göttingen, Göttingen, Deutschland; 9 UroEvidence, Deutsche Gesellschaft für Urologie (DGU), Berlin, Deutschland

**Keywords:** Harnblasenkarzinom, Bacillus Calmette-Guérin, Frühzystektomie, Überleben, Geschlecht, Bladder cancer, Bacillus Calmette-Guérin, Early cystectomy, Survival, Gender

## Abstract

**Hintergrund:**

Trotz geringerer Inzidenz zeigen Frauen mit Harnblasenkarzinom schlechtere Überlebensraten als Männer. Bisherige Studien liefern jedoch nur unzureichende Daten zu geschlechtsspezifischen Therapieergebnissen, insbesondere nach radikaler Frühzystektomie bei BCG-Versagen (Bacillus Calmette-Guérin).

**Fragestellung:**

Gibt es geschlechtsspezifische Unterschiede im onkologischen Outcome und in der Komplikationsrate nach radikaler Frühzystektomie bei Patienten mit nicht-muskelinvasivem Harnblasenkarzinom nach Versagen der BCG-Instillationstherapie?

**Material und Methoden:**

Im Januar 2025 wurde eine systematische Literaturrecherche in MEDLINE und der Cochrane Library durchgeführt. Das vollständige Studienprotokoll ist prospektiv bei PROSPERO registriert (ID CRD42024611111) worden.

**Ergebnisse:**

Drei retrospektive Kohortenstudien mit insgesamt 655 Patient*innen (davon 18,8 % Frauen) wurden eingeschlossen. Es zeigten sich keine signifikanten geschlechtsspezifischen Unterschiede in Bezug auf Gesamtüberleben, krebsspezifisches Überleben oder Progression. Daten zu Komplikationen und Lebensqualität lagen nur begrenzt bzw. nicht vor. In 2 Studien wurde der Befall der prostatischen Urethra als negativer prognostischer Marker bei Männern identifiziert.

**Schlussfolgerung:**

Die aktuelle Evidenzlage ist spärlich, methodisch limitiert und durch die Unterrepräsentation von Frauen verzerrt. Daher sind belastbare Aussagen zur Auswirkung des Geschlechts auf die Wirksamkeit der Frühzystektomie nach BCG-Versagen nicht möglich. Weitere Forschung zu geschlechtsabhängigen Risikofaktoren, insbesondere hormonellen und immunologischen Einflüssen, ist zur Entwicklung von personalisierten Therapiestrategien dringend erforderlich.

## Abstract (en)

**Background:**

Despite a lower incidence, women with bladder cancer have poorer survival rates than men. However, studies to date have provided insufficient data on gender-specific treatment outcomes, particularly after radical early cystectomy for Bacillus Calmette-Guérin (BCG) failure.

**Objectives:**

Are there gender-specific differences in oncologic outcomes and complication rates after radical early cystectomy in patients with nonmuscle invasive bladder cancer after failure of BCG instillation therapy?

**Materials and methods:**

A systematic literature search was conducted in MEDLINE and the Cochrane Library in January 2025. The complete study protocol has been prospectively registered at PROSPERO (ID CRD42024611111).

**Results:**

Three retrospective cohort studies with a total of 655 patients (18.8% of whom were women) were included. There were no significant gender-specific differences in terms of overall survival, cancer-specific survival, or progression. Data on complications and quality of life were limited or not available. In two studies, prostatic urethral involvement was identified as a negative prognostic marker in men.

**Conclusion:**

The current evidence base is sparse, methodologically limited, and biased by the underrepresentation of women. Therefore, it is not possible to make reliable statements on the effect of gender on the efficacy of early cystectomy after BCG failure. Further research on gender-dependent risk factors, especially hormonal and immunological influences, is urgently needed to develop personalized treatment strategies.

## Hintergrund und Fragestellung

Harnblasenkrebs ist bei Männern die siebthäufigste diagnostizierte Krebsart. Frauen hingegen sind deutlich seltener betroffen: In der Europäischen Union beträgt die altersstandardisierte Inzidenzrate 20 für Männer (pro 100.000 Einwohner/pro Jahr) und 4,6 für Frauen. Weltweit lag die altersstandardisierte Harnblasenkrebs-Sterblichkeitsrate (pro 100.000 Einwohner/pro Jahr) 2012 bei 3,3 für Männer und 0,86 für Frauen [1]. Die Inzidenz- und Sterblichkeitsraten von Harnblasenkrebs variieren länderspezifisch aufgrund von Unterschieden bei den Risikofaktoren, der Erkennungs- und Diagnosepraxis und der Verfügbarkeit von Therapien [2, 3].

Obwohl Männer häufiger an Harnblasenkrebs erkranken als Frauen, ist die Krankheit bei Frauen oft weiter fortgeschritten und die Überlebensraten sind schlechter. Eine Metaanalyse mit fast 28.000 Patienten zeigte, dass das weibliche Geschlecht mit einem schlechteren Überleben (HR = 1,20; 95 %-KI 1,09–1,32) im Vergleich zum männlichen Geschlecht nach radikaler Zystektomie verbunden war [4]. Es ist jedoch unwahrscheinlich, dass die Unterschiede im Gesamtüberleben (OS) durch allein komplexe Behandlungsmuster erklärt werden können [5]. Eine bevölkerungsbezogene Studie aus den MarketScan-Datenbanken deutet darauf hin, dass ein möglicher Grund für die schlechteren Überlebenschancen in der weiblichen Bevölkerung darin liegen könnte, dass bei Frauen die Diagnose länger hinausgezögert wird als bei Männern, da die Differentialdiagnose bei Frauen Krankheiten wie z. B. Harnwegsinfektionen umfasst, die häufiger auftreten als Harnblasenkarzinome selbst [6]. Darüber hinaus können die Unterschiede in der geschlechtsspezifischen Prävalenz von Harnblasenkarzinomen neben Tabak- und Chemikalienexposition auch auf weitere Faktoren zurückzuführen sein. In einer großen prospektiven Kohortenstudie wurde der postmenopausale Status mit einem erhöhten Harnblasenkrebsrisiko in Verbindung gebracht, selbst nach Anpassung für einen Raucherstatus. Dieses Ergebnis könnte daraufhin deuten, dass die Unterschiede im Östrogen- und Androgenspiegel zwischen Männern und Frauen für einen Teil der Unterschiede in der geschlechtsspezifischen Prävalenz von Harnblasenkarzinomen verantwortlich sind [7–9]. Eine relativ aktuelle Bevölkerungsstudie, in der die Auswirkungen der Hormone auf das Harnblasenkarzinom untersucht wurden, legt zudem nahe, dass ein jüngeres Alter bei der Menopause (≤45 Jahre) ebenfalls mit einem erhöhten Harnblasenkrebsrisiko verbunden ist [10].

Allerdings bleibt weiterhin unklar, welche Faktoren in welchem Umfang konkret zum schlechteren Outcome von Patientinnen bei dieser Tumorerkrankung beitragen. Ein möglicher Ansatz ist die spezifische Analyse einzelner möglicher Faktoren bzw. einzelner Therapien, inklusive des Versuchs möglichst viele Confunding-Faktoren auszuschalten. Aus diesem Grund hat unsere Arbeitsgruppe bisher 3 systematische Übersichtsarbeiten mit Metaanalyse durchgeführt, um den Einfluss einzelner Therapien auf geschlechtsspezifische Unterschiede beim Harnblasenkarzinom zu bewerten. In einer ersten Analyse evaluierten wir den Einfluss von Immuntherapien beim metastasierten bzw. fortgeschrittenen Harnblasenkarzinom und kamen zu der Schlussfolgerung, dass es eine Tendenz für bessere Ergebnisse bei den Frauen gibt, aber nur der Antikörper Atezolizumab zeigt eine signifikant besser Gesamtansprechrate bei Patientinnen. Leider werden in vielen Studien keine geschlechtsspezifischen Ergebnisse berichtet. Daher ist weitere die Forschung unerlässlich, wenn man eine individualisierte medizinische Versorgung anstrebt. Diese Forschung sollte sich zusätzlich mit immunologischen Störfaktoren befassen [11]. In einem zweiten Schritt untersuchten wir deshalb die Unterschiede bei der BCG-Instillationstherapie (Bacillus Calmette-Guérin) beim nicht-muskelinvasiven Harnblasenkarzinom (NMIBC). Die Ergebnisse zeigen keinen Zusammenhang zwischen Geschlecht und onkologischen Ergebnissen nach BCG [12]. Aus diesem Grund analysierten wir schließlich die Unterschiede bei der adjuvanten zytostatischen, intravesikalen Therapie beim NMIBC. In dieser Analyse wurden zwar keine signifikanten geschlechtsspezifischen Unterschiede bei den Therapieergebnissen von NMIBC nach intravesikaler Chemotherapie festgestellt, doch sind die Ergebnisse durch die geringe Anzahl von Studien, die Unterrepräsentation von Frauen und die inkonsistente Berichterstattung bzgl. kritischer Ergebnisse begrenzt. Künftige Forschungsarbeiten sollten geschlechtsspezifische Analysen in den Vordergrund stellen und die molekularen und genetischen Grundlagen potenzieller Unterschiede untersuchen, um die Präzisionsmedizin und eine gerechte Versorgung zu unterstützen [13].

Um sich der Problemstellung weiter zu nähern und ggf. einzelne Einflussfaktoren auf das beschriebene schlechtere Outcome zu identifizieren, um schlussendlich eine verbesserte und individualisierte Therapie für Frauen bei dieser Tumorerkrankung zu ermöglichen, widmeten wir uns schließlich folgender Frage im Rahmen einer systematischen Übersichtsarbeit: Gibt es geschlechtsspezifische Unterschiede in Bezug auf das Ergebnis und die Überlebenschancen einer Frühzystektomie bei Patienten, bei denen eine BCG-Instillationstherapie bei Harnblasenkarzinom fehlgeschlagen ist?

Das primäre Ziel dieser Arbeit ist dabei, geschlechtsspezifische Unterschiede in der Komplikationsrate (während der stationären Behandlung, nach 30 und 90 Tagen), im rezidivfreien (RFS), progressionsfreien (PFS), krebsspezifischen (CSS) und Gesamtüberleben (OS) bei diesen Patienten zu bewerten. Das sekundäre Ziel ist die Bewertung von geschlechtsspezifischen Unterschieden in der Lebensqualität (QoL), sofern diese Daten vorhanden sind.

## Methodik

Im Januar 2025 führten wir eine systematische Literaturrecherche in MEDLINE via PubMed und im Cochrane Library durch, um die von uns definierten Fragestellungen zu beantworten. Das Protokoll dieser systematischen Übersichtsarbeit inklusive der Suchstrategien wurde dabei prospektiv in der Plattform PROSPERO registriert (https://www.crd.york.ac.uk/prospero/; ID CRD 42024611111). Dabei ist das vollständige Review-Protokoll unter der entsprechenden Registrierungsnummer einsehbar. Insgesamt wurden keine Einschränkungen hinsichtlich der Sprache, der Studienregion oder der Art der Veröffentlichung gemacht.

Hinsichtlich der Patientenpopulation sollten Erwachsene mit Harnblasenkarzinom, die eine TURB erhalten haben sowie nachfolgend eine adjuvante BCG-Instillationstherapie, die dann versagt hat und schließlich eine radikale Zystektomie erhalten haben, eingeschlossen werden. Daher handelt es sich bei der Intervention um die radikale Zystektomie mit Harnableitung bei BCG-Versagen. Die Kombination mit einer Strahlentherapie oder mit der Instillation gezielter, zytostatischer oder immunmodulierender Therapien sollte nicht berücksichtigt werden, um Confounding-Faktoren zu minimieren.

Für jeden eingeschlossenen Datensatz wurde ein a priori festgelegtes standardisiertes Datenextraktionsverfahren verwendet, die prädefinierten Variablen sind dabei ebenfalls über das PROSPERO-Protokoll einsehbar. Die Studienextraktion wurde unabhängig voneinander von 2 Autoren durchgeführt. Unstimmigkeiten wurden von einem dritten Review-Autor geklärt. Die Online-Plattform covidence (https://www.covidence.org/; Veritas Health Innovation Ltd, Melbourne, Australien) wurde für das Screening und die Datenextraktion verwendet.

Zwei Autoren bewerteten unabhängig voneinander das Risiko einer Verzerrung mit dem ROBINS-I-Instrument. Das ROBINS-I-Instrument umfasst 7 Bereiche der Verzerrung: Risiko der Verzerrung aufgrund von Confounding, Verzerrung bei der Auswahl der Studienteilnehmer, Verzerrung bei der Klassifizierung der Interventionen, Verzerrung aufgrund von Abweichungen von den geplanten Interventionen, Verzerrung aufgrund fehlender Daten, Verzerrung bei der Messung der Ergebnisse und Verzerrung bei der Auswahl der berichteten Ergebnisse für eine Ergebnismessung. Die Bereiche werden zu einem Gesamtrisiko für Bias zusammengefasst [14]. Etwaige Unstimmigkeiten wurden durch die Einbeziehung eines dritten Autors dieser systematischen Übersichtsarbeit gelöst.

Der Vergleich der geschlechtsspezifischen Unterschiede bei den Studienzielen sollte mit der Methode der inversen Varianzgewichtung für die Zusammenführung kontinuierlicher Ergebnisdaten durchgeführt werden, wie im PROSPERO-Protokoll beschrieben. Allerdings konnte aufgrund der Heterogenität der inkludierten Studien kein Daten-Pooling vorgenommen werden, so dass die Darstellung nun rein deskriptiv erfolgt.

## Ergebnisse

**Abb. 1:**
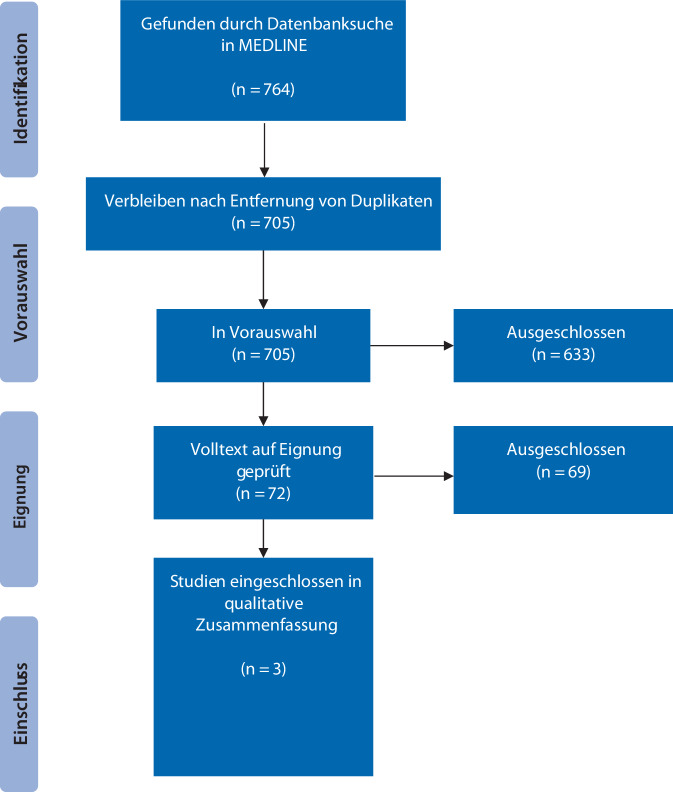
PRISMA-Flussdiagramm

Die primäre Literatursuche ergab 764 Treffer, letztendlich konnten 3 retrospektive Kohortenstudien mit insgesamt 655 Patienten, davon 123 (18,8 %) Patientinnen, in die Evidenzsynthese eingeschlossen werden (Abb. 1: PRISMA-Flussdiagramm). Gründe für den Ausschluss von Studien im Volltext-Screening waren: 39 Studien haben keine geschlechtsspezifische Analyse durchgeführt, 27 hatten das falsche Studien-Setting, 2 Studien das falsche Studiendesign und 1 Studie berichtet über nicht für unsere systematischer Übersichtsarbeit relevante Ergebnisse.

In Tab. 1 sind die Hauptergebnisse der inkludierten Studien dargestellt [15–17]. Zwei Untersuchungen wurden dabei in den USA durchgeführt und eine in Spanien. Alle 3 Studien sind in hochrangigen englisch-sprachigen Journalen als Volltext publiziert worden. Weiterhin wurden die inkludierten Analysen zwischen 2005 und 2016 veröffentlicht.

**Tab. 1:** Hauptcharakteristika der eingeschlossenen Studien (*n* = 3)

Referenz	Studiendesign	Geschlechtsspezifische Unterschiede als Studienziel	Patientenpopulation	Hauptergebnisse geschlechtsspezifische Unterschiede	Autorenschlussfolgerungen
*Haas et al., 2016 *[15]	Retrospektive Kohortenstudie	Nein	117 Patienten, die sich zwischen 1990 und 2012 unizentrisch einer radikalen Zystektomie aufgrund von rezidivierenden NMIBC unterziehen mussten; die Kohorte wurde in zwei Gruppen aufgeteilt: Gruppe 1 (*n* = 61) bestand aus Patienten, die nur mit BCG ± Interferon‑α behandelt wurden; Gruppe 2 (*n* = 56) erhielt mindestens eine zusätzliche intravesikale Chemotherapie nach BCG; 30 Patientinnen (25,6 %) in der Gesamtkohorte	In der univariaten Cox-Regressionsanalyse zu OS und CSS ist das weibliche Geschlecht kein Risikofaktor (HR = 0,62; 95 %-KI 0,32–1,24; *p* = 0,117)	Bei geeigneter Auswahl der Patienten für eine intravesikale Chemotherapie zur Wiederherstellung der Blasenfunktion müssen Patienten, die sich für eine blasenschonende Behandlung anstelle einer früheren radikalen Zystektomie nach Versagen der BCG entscheiden, keine Abstriche bei den pathologischen oder onkologischen Ergebnissen machen, während die Blasenfunktion für eine deutlich längere Zeit erhalten bleibt
*Chade et al., 2010 *[16]	Retrospektive Kohortenstudie	Nein	476 Patienten mit High-grade-cTis, darunter 221 mit primärem und 255 mit sekundärem CIS, von 1990 bis 2008, nach transurethraler Resektion und intravesikaler BCG-Therapie; 87 Patientinnen (18,3 %) in der Gesamtkohorte	In der multivariaten Analyse der Progression zu cT1 oder höherer invasiver Erkrankung und cT2 oder höherer invasiver Muskelerkrankung, bereinigt um RC vor der Progression ist das Geschlecht sowohl für cT1 (HR 1,18; 95 %-KI 0,86–1,63; *p* = 0,300) als auch für cT2 (HR 1,15; 95 %-KI 0,80–1,65; *p* = 0,455) kein Risikofaktor	Patienten mit einem primären CIS haben ein schlechteres Ergebnis als Patienten mit einem sekundären CIS, was darauf hindeutet, dass diese beiden Entitäten bei der Behandlungsentscheidung unterschieden werden müssen
*Huguet et al., 2005 *[17]	Retrospektive Kohortenstudie	Nein	62 Patienten mit NMIBC pTis, pTa und pT1 bei denen eine Therapie mit TURB und BCG versagte und die Indikation zur radikalen Zystektomie bestand; 6 Patientinnen (9,7 %) in der Gesamtkohorte	In der multivariaten Analyse der klinisch-pathologischen Faktoren, die mit einem unterschätzendem Staging zusammenhängen, war das Geschlecht (HR 0,1; 95 %-KI 0,01–1,5; *p* = 0,1) kein Risikofaktor	Die RC sollte bei oberflächlichen Tumoren mit hohem Risiko, die nach TUR und BCG versagen, vor der Progression durchgeführt werden. Bei Patienten mit klinischer und pathologischer nicht-muskelinvasiver Erkrankung bietet die RC ein ausgezeichnetes krankheitsfreies Überleben. Tumor in der prostatischen Urethra war der einzige Risikofaktor für unterschätztes Staging und schlechteres Überleben

*NMIBC* nicht-muskelinvasiver Blasenkrebs, *OS* Gesamtüberleben, *CSS* krebsspezifisches Überleben, *KI* Konfidenzintervall, *HR* Hazard Ratio, *CIS* Carcinoma in situ, *BCG* Bacillus Calmette-Guérin

### Geschlechtsspezifische Unterschiede

Das initiale primäre Studienziel war es, geschlechtsspezifische Unterschiede in der Komplikationsrate (während der stationären Behandlung, nach 30 und 90 Tagen), im RFS, PFS, CSS und OS bei diesen Patienten zu bewerten. Allerdings ist die Datenlage selbst in den inkludierten Studien extrem spärlich, insbesondere was konkrete Ergebnisse nach der radikalen Frühzystektomie betrifft. Haas et al. berichten, dass in der univariaten Cox-Regressionsanalyse für OS und CSS das weibliche Geschlecht keinen Risikofaktor darstellt (HR = 0,62; 95 %-KI 0,32–1,24; *p* = 0,117; [15]). Weiterhin stellt in der Arbeit von Chade et al. das Geschlecht keinen Risikofaktor für eine Progression im Staging dar sowie keinen Risikofaktor für unterschätztes Staging in der Studie von Huguet et al. [16, 17]. Daten zu den Komplikationsraten während des stationären Aufenthaltes liegen nicht vor.

Das initiale sekundäre Studienziel dieser Arbeit waren geschlechtsspezifische Unterschiede hinsichtlich der Lebensqualität. Hierzu liegen in den inkludierten Studien ebenfalls keine Daten vor.

Interessanterweise berichten 2 der eingeschlossenen Arbeiten, dass der Befall der prostatischen Harnröhre ein signifikanter Risikofaktor bei BCG-Versagern für schlechtere Ergebnisse ist, so schlussfolgern Huguet et al.: Das Vorhandensein von Tumor in der prostatischen Harnröhre zum Zeitpunkt des endoskopischen Stagings vor der radikalen Zystektomie war der einzige Faktor, der mit einem klinischen unterschätztem Staging (*p* = 0,003) und einem kürzeren Überleben (*p* < 0,0002) signifikant verbunden war. Aus diesem Ergebnis schlussfolgerten die Autoren, dass dies darauf zurückzuführen sei, dass ein korrektes endoskopisches Staging der prostatischen Urethra schwierig ist und bei Patienten, die BCG erhalten haben, die prostatische Harnröhre ein Ort ist, an dem ein Urothelkarzinom unbemerkt fortschreiten kann [17]. Haas et al. berichten zusätzlich, dass der Befall der prostatischen Harnröhre signifikant mit einem schlechteren Gesamtüberleben nach der radikalen Zystektomie assoziiert ist (HR = 1,95; 95 %-KI 1,07–3,54; *p* = 0,029; [15]).

### Qualitätsbewertung der inkludierten Studien

Die Tab. 2 illustriert die Qualitätsbewertung der 3 eingeschlossenen Studien. Insgesamt liegt ein moderates Risiko für Verzerrungen vor, vorrangig aufgrund des retrospektiven Studiendesigns mit Selektionsbias.

**Tab. 2:** Qualitätsbewertung der inkludierten Studien mit dem ROBINS-I-Instrument (*n* = 3)

Referenz	Risk of bias due to confounding	Bias in selection of participants into the study	Bias in classification of interventions	Bias due to deviations from intended interventions	Bias due to missing data	Bias in measurement of outcomes	Bias in selection of the reported results	Gesamtrisiko für Bias
*Haas et al., 2016 *[15]	Moderates Risiko	Moderates Risiko	Niedriges Risiko	Moderates Risiko	Moderates Risiko	Niedriges Risiko	Niedriges Risiko	*Moderates Risiko*
*Chade et al., 2010 *[16]	Moderates Risiko	Moderates Risiko	Niedriges Risiko	Niedriges Risiko	Moderates Risiko	Niedriges Risiko	Niedriges Risiko	*Moderates Risiko*
*Huguet et al., 2005 *[17]	Moderates Risiko	Moderates Risiko	Niedriges Risiko	Niedriges Risiko	Niedriges Risiko	Niedriges Risiko	Niedriges Risiko	*Moderates Risiko*

## Diskussion

Wir führten eine systematische Übersichtsarbeit zu geschlechtsspezifischen Unterschieden bei den Ergebnissen nach radikaler Frühzystektomie durch, um einen spezifischen Faktor zu identifizieren, der zu den berichteten schlechteren Ergebnissen bei Harnblasenkarzinomen bei Patientinnen beiträgt. Leider ist die Datenlage extrem spärlich und es ergeben sich hier keine Hinweise, dass Frauen bei der Frühzystektomie schlechtere Ergebnisse erleiden. Doch es muss zusätzlich angemerkt werden, dass sich bei den eingeschlossenen Studien ausschließlich um retrospektive Kohorten mit moderater Studienqualität handelt. Hervorzuheben als Beispiel für die moderate Qualität ist der Fakt, dass Haas et al. eine Cox-Regressionsanalyse für OS und CSS zusammen durchgeführt haben [15]. Hier muss betont werden, dass insbesondere in der onkologischen Forschung auf korrekte Definitionen, Begrifflichkeiten und Berichterstattung zu achten ist und insbesondere in einer älteren Studienpopulation CSS und OS nicht unbedingt identisch sind. Außerdem hatte keine der inkludierten Studien die Deskription von geschlechtsspezifischen Unterschieden als Studienziel.

In der Literatur gibt es aus einer multivariaten Regressionsanalyse zusätzlich keinen Hinweis darauf, dass das Geschlecht bei pT1-High-grade-Harnblasenkarzinomen keinen Einfluss auf die Durchführung einer radikalen Frühzystektomie hat (OR 1,05; 95 %-KI 0,89–1,22; *p* = 0,546; [18]). Allerdings merken auch hier die Autoren an, dass zahlreiche Confounding-Faktoren Einfluss auf die Ergebnisse der Frühzystektomie haben können, z. B. die BCG-Lieferengpässe, die Anwesenheit von Carcinoma in situ, die Anzahl der durchgeführten transurethralen Resektionen sowie BCG-Instillationen und eben auch, wie für die weibliche Population beschrieben, der Zeitpunkt der Diagnosestellung. Weiterhin ist ebenfalls entscheidend, welche konkrete Definition von BCG-Versagen verwendet worden ist [16, 18].

Interessanterweise wird in 2 der inkludierten Studien berichtet, dass der Befall der prostatischen Harnröhre einen negativen prognostischen Faktor darstellt [15, 17]. Dies bedeutet wiederum, ein schlechteres Ergebnis für die betroffenen Männer und lenkt die Diskussion auf die Bedeutung von Androgenrezeptoren sowie von Östrogen- und Androgenspiegeln beim Harnblasenkarzinom. In ihrer umfassenden Übersichtsarbeit beschreiben Dotto et al., dass die Rolle des Androgenrezeptors in der Harnblasenkrebsprogression komplex zu sein scheint, da verschiedene Studien in diesem Zusammenzusammenhang sowohl erhöhte als auch erniedrigte Androgenrezeptorspiegel berichten. Diese Diskrepanz könnte die Heterogenität der Androgenrezeptorexpression im Zusammenhang mit der Vielfalt und Plastizität der Harnblasenkrebssubtypen sowie die Rolle des Androgenrezeptors im Tumormikromilieu wiederspiegeln. Auf molekularer Ebene sind dabei multiple Mechanismen beteiligt, z. B. der Androgenrezeptor-EGFR-Crosstalk und die androgenrezeptorabhängige transkriptionelle Hochregulierung von CD24 [19]. Daher sollten diese Zusammenhänge in zukünftigen translationalen als auch klinischen Studien weiter erforscht werden, um eine hochspezialisierte und individualisierte Therapie zu ermöglichen. Doch auch der Einfluss der Geschlechtshormone auf das Immunsystem könnte ein entscheidender Faktor sein. Interessanterweise haben regulatorischen B‑Zellen eine antiinflammatorische Wirkung. Darüber hinaus haben ältere Studien gezeigt, dass Östrogene regulatorische B‑Zellen hochregulieren können und damit indirekt eine entzündungshemmende Wirkung haben. Einige Autoren schließen daraus, dass sich autoimmunvermittelte Krankheiten wie multiple Sklerose während der Schwangerschaft klinisch bessern oder nicht fortschreiten [20–26]. Folglich kann nun davon ausgegangen werden, dass die entzündungshemmenden regulatorischen B‑Zellen bei Frauen aufgrund des Vorhandenseins von Östrogen stärker exprimiert werden als bei Männern und dass die entzündungshemmende Wirkung einen Unterschied bei der Kontrolle von Harnblasenkarzinomen ausmachen kann [13]. Hier bedarf es ebenfalls weiterer Forschung, bei der auch der prä- bzw. postmenopausale Status der Frau mit in Betracht gezogen werden muss.

Kritisch ist zu diskutieren, dass gerade Studien zu Harnblasenkarzinomen nur unzureichend Komplikationen sowie Daten zur Lebensqualität berichten, geschlechtsspezifische Daten liegen fast gar nicht vor. Bereits in unseren Vorarbeiten hat unsere Arbeitsgruppe angemerkt, dass eine umfassende Berichterstattung über unerwünschte Ereignisse und Lebensqualität integraler Bestandteil des Studiendesigns sein sollte, um ein ganzheitliches Verständnis der Auswirkungen der Behandlung zu ermöglichen und eine patientenzentrierte Versorgung zu gewährleisten [11–13].

Diese Arbeit ist ebenfalls nicht ohne Limitationen, insbesondere durch die kleine Anzahl der eingeschlossenen Studien und der rein deskriptiven Darstellung der Ergebnisse. Weiterhin sind Frauen in dieser Arbeit mit 18,8 % unter repräsentiert, da beim Harnblasenkarzinom ein Anteil von etwa 25 % weiblicher Patienten zu erwarten wäre. Aus diesen Gründen kann die Frage, ob Frauen bei der Frühzystektomie schlechtere Ergebnisse erleiden aus der aktuellen Datenlage nicht abschließend beantwortet werden.

Trotzdem bleibt die Frage, welche Faktoren in welchem Ausmaß zu den gut dokumentierten schlechteren Ergebnissen von Patientinnen mit Harnblasenkarzinom führen. Die Beantwortung dieser Frage ist ein essentieller Schritt zur individualisierten Medizin mit hochspezifischen Therapien und damit schlussendlich zur Verbesserung unserer Therapieergebnisse. Dazu ist weitere qualitative Forschung im Bereich der Geschlechtermedizin notwendig. Außerdem sollten künftige Forschungsarbeiten über binäre Geschlechtervergleiche hinausgehen, z. B. das Spektrum geschlechtsspezifischer Effekte untersuchen, einzelne geschlechtsspezifische Faktoren isolieren, um Verwechslungen zu vermeiden, potenzielle Wechselwirkungen zwischen Faktoren bewerten und geschlechtsspezifische, altersbedingte Veränderungen wie den Rückgang von Hormonen berücksichtigen [27]. Zusätzlich müssen in dieser weiteren Forschung zahlreiche Confounder beachtet werden, wie z. B. die Assoziation von Diabetes-mellitus-Medikation mit der Entstehung und Progression von Harnblasenkrebs [28]. Diese spezifische Forschung lässt sich aus unserer Sicht durch 3 Ansätze gezielt weiterverfolgen:

1. Datenintegration und künstliche Intelligenz wie z. B. auch in der Netzwerkmetaanalyse von Cerrato et al. beschrieben [29]. Dies ist notwendig um komplexe Zusammenhänge zu begreifen und eine komplexe Risikoeinschätzung zu treffen, aber auch um einzelne spezifische Faktoren zu identifizieren, die dann weiter untersucht werden können.
2. Robuste Metaanalysen und klinische Studien zu Evaluierung einzelner Faktoren unter Ausschluss möglichst vieler Störfaktoren.
3. Translationale Erforschung von Hormonstatus und Immunsystem im Zusammenhang mit Harnblasenkarzinomen. Aufgrund der aktuellen Datenlage scheinen hier die Evaluation des Androgenrezeptors und regulatorische B‑Zellen besonders sinnvoll.

Unsere Arbeitsgruppe fokussiert sich dabei aktuell auf den zweiten Ansatz und plant nun in einem nächsten Schritt eine systematische Übersichtsarbeit mit Metaanalyse zu geschlechtsspezifischen Unterschieden bei der Therapie mit Enfortumab-Vedotin beim metastasierten Harnblasenkarzinom. Das Review-Protokoll ist bereits prospektiv registriert und über PROSEPERO verfügbar (https://www.crd.york.ac.uk/prospero/; ID CRD 420251064260). Eine weitere Fragestellung, die sich aus diesem Kontext ergibt, ist, was geschlechtsspezifische Unterschiede sind bzw. ob es diese gibt, wenn BCG-Versager blasenerhaltend therapiert werden. Hier erwarten wir die Vollpublikation der CISTO-Studie, deren erste Daten auf dem AUA 2025 vorgestellt worden sind [30].

## Fazit für die Praxis

- Frauen mit Harnblasenkarzinom haben insgesamt schlechtere Therapieergebnisse als Männer.
- Bezüglich der radikalen Frühzystektomie nach BCG-Versagen (Bacillus Calmette-Guérin) zeigt sich aufgrund der eingeschränkten Datenmenge und -qualität kein signifikanter Geschlechterunterschied.
- Der Befall der prostatischen Urethra bei Männern stellt einen gesicherten negativen Prognosefaktor dar.
- Die weitere Erforschung geschlechtsspezifischer Unterschiede ist ein essentieller Schritt zur individualisierten Medizin und zur Verbesserung der Therapieergebnisse.
- Klinische Studien sollten konsequent und detailliert über Komplikationsraten und Lebensqualität berichten.
- Translationale Studien zur Rolle von Sexualhormonen und zu geschlechtsspezifischen immunologischen Faktoren sind dringend anzustreben.
